# Effects of Chemically-Functionalized Single-Walled Carbon Nanotubes on the Morphology and Vitality of D54MG Human Glioblastoma Cells

**DOI:** 10.3390/neuroglia1020022

**Published:** 2018-10-16

**Authors:** Seantel Hopkins, Manoj K. Gottipati, Vedrana Montana, Elena Bekyarova, Robert C. Haddon, Vladimir Parpura

**Affiliations:** 1 Department of Biology, The University of Alabama at Birmingham, Birmingham, AL 35294, USA; 2 Department of Neurobiology, The University of Alabama at Birmingham, Birmingham, AL 35294, USA; 3 Department of Biomedical Engineering and Center for Biotechnology and Interdisciplinary Studies, Rensselaer Polytechnic Institute, Troy, NY 12180, USA; 4 Department of Neuroscience, Center for Brain and Spinal Cord Repair and Wexner Medical Center, The Ohio State University, Columbus, OH 43210, USA; 5 Departments of Chemistry and Chemical Engineering and Center for Nanoscale Science and Engineering, University of California, Riverside, CA 92521, USA; 6 Carbon Solutions, Inc., Riverside, CA 92507, USA

**Keywords:** glioblastoma multiforme, carbon nanotubes, morphology, cell adhesion, cell proliferation, cell death rate

## Abstract

The unique properties of single-walled carbon nanotubes (SWCNTs) have made them interesting candidates for applications in biomedicine. There are diverse chemical groups that can be attached to SWCNTs in order for these tiny tubes to gain various functionalities, for example, water solubility. Due to the availability of these “functionalization” approaches, SWCNTs are seen as agents for a potential anti-cancer therapy. In this context, we tested different chemically-functionalized forms of SWCNTs to determine which modifications make them better combatants against glioblastoma (astrocytoma grade IV), the deadliest brain cancer. We investigated the effects that two types of water soluble SWCNTs, functionalized with polyethylene glycol (SWCNT-PEG) or tetrahydrofurfuryl-terminated polyethylene glycol (SWCNT-PEG-THFF), have on the morphology and vitality, that is, cell adhesion, proliferation and death rate, of the D54MG human glioblastoma cells in culture. We found that SWCNT-PEG-THFF solute, when added to culture media, makes D54MG cells less round (measured as a significant decrease, by ~23%, in the form factor). This morphological change was induced by the PEG-THFF functional group, but not the SWCNT backbone itself. We also found that SWCNT-PEG-THFF solute reduces the proliferation rate of D54MG cells while increasing the rate of cell death. The functional groups PEG and PEG-THFF, on the other hand, reduce the cell death rate of D54MG human glioma cells. These data indicate that the process of functionalization of SWCNTs for potential use as glioma therapeutics may affect their biological effects.

## 1. Introduction

Gliomas comprise the large majority of malignant brain tumors and are one of the deadliest cancers, with a median survival of 14 months. The most aggressive glioma type is classified, based on its cell origin, as astrocytoma (high) grade IV, traditionally referred to as a glioblastoma multiforme (GBM), which is the subject of this work. High grade gliomas are characterized by extensive dispersal throughout the brain, indicative of their highly invasive nature [1]. Glioma cells have to adjust their morphology, that is, become less round, during their invasion through the narrow and tortuous extracellular space of the brain [2]. Aside from their invasive nature, their adhesion and proliferative capabilities are factors contributing to their malignancy [3,4]. Finding new treatments that would stop/attenuate the spread of GBMs would be a milestone; this requires a detailed analysis of GBM biology.

The unique properties of single-walled carbon nanotubes (SWCNTs) have made them interesting candidates for applications in biotechnology and biomedicine [5,6]. In the context of glioma nanotechnology and translational therapeutics, functionalized SWCNTs have been used as carriers in advanced drug delivery systems [7–9] or as agents for photothermal therapy [10–13] in variety of human GBM and murine models, capitalizing on the availability of diverse and well established chemistries for the functionalization of SWCNTs [5,6] (i.e., attachment of various chemicals to the SWCNTs in order for tubes to gain a functionality, e.g., water solubility) and their properties to produce heat when exposed to non-ionizing near-infrared radiation [14]. These investigations have driven the development of functionalized SWCNTs as a carrier/delivery agent to cancer tissue; a variety of functionalizations of SWCNTs with molecules rendering their water solubility, a prerequisite for bio applications, have been developed. However, there has been a void in understating the effect of the components of such conjugate nanomaterials on GBM cells. For instance, possible effects of the functionalization group(s) of the conjugate SWCNT nanomaterial are not well described in the literature. Similarly, basic cell biology measurements, such as cell morphology, adhesion, proliferation and death rate, caused by the conjugate SWCNTs vs. their solubilization functional groups, have not been well described. In the present work we set to investigate these issues.

Our present work logically sprouts from our previous studies of the effect that SWCNTs have on primary astrocytes, as reviewed elsewhere [15]. Briefly, to investigate the effects of SWCNTs on primary mouse astrocytes in culture, we used graft copolymers/conjugates of SWCNTs chemically functionalized with polyethylene glycol (PEG) or poly-m-aminobenzene sulfonic acid (PABS) making SWCNTs water soluble (wsSWCNTs) [16,17]. When added to the culturing medium, wsSWCNTs were able to make astrocytes larger and less round compared to the untreated astrocytes, but these nanomaterials did not affect astrocyte vitality, that is, cell adhesion, proliferation and death rate [17]. Generally, the data indicated the necessity of the SWCNT backbone for the changes induced by the water-soluble graft copolymers, while some subtle differences in the effects that SWCNTs had on astrocytes (for clarity details omitted here, but summarized and reviewed in [15] and Table 1 of [17]; also see discussion) were due to the various functional groups attached to the SWCNTs.

In the present study, we aim to investigate the effects that two types of wsSWCNTs, SWCNTs functionalized with polyethylene glycol (SWCNT-PEG) [16,17] and SWCNTs functionalized with tetrahydrofurfuryl-terminated polyethylene glycol (SWCNT-PEG-THFF) [18], have on the morphology and vitality of the D54MG human glioma cell line. We found that SWCNT-PEG-THFF solute induces morphological changes in D54MG human glioma cells. These changes were induced by the functional group, and not the SWCNT backbone itself. Other findings show that, SWCNT-PEG-THFF solute reduces the proliferation potential of D54MG human glioma along with increasing the relative cell death rate, while the functional groups PEG and PEG-THFF reduce the cell death rate of D54MG human glioma cells. Taken together, our present work indicates that additional care should be taken in the process of functionalization of SWCNTs for potential use as glioma therapeutics, as SWCNT conjugates may cause differential biological effects pending on the functional group rendering their water solubility.

## 2. Materials and Methods

### 2.1. Water-Soluble Single-Walled Carbon Nanotubes

Single-walled carbon nanotubes were rendered water soluble by covalent attachment of PEG (molecular mass 600 g/mol) or PEG-THFF (molecular mass 200 g/mol) to their walls. These graft copolymers were synthesized and characterized as described in detail elsewhere [16,18]. Single-walled carbon nanotubes- polyethylene glycol used in this study contained, 23 wt% PEG, while the SWCNT-PEG-THFF contained 21 wt% PEG-THFF. Both the SWCNT graft co-polymers were used as colloidal aqueous solutes in cell culture media (see below) at a concentration of 5 μg/mL, chosen based on our previous work [16,17,19]. The functional groups, PEG or PEG-THFF, were used at a concentration of 1 μg/mL, corresponding to 20 wt% of the functionalized wsSWCNTs.

### 2.2. Human Glioblastoma Multiforme Cell Culture

The human glioma cell line D54MG (classified as a glioblastoma multiforme, GBM, also known as grade IV astrocytoma) modified to stably express enhanced green fluorescent protein (EGFP), as previously described [20,21], was provided by Dr. Harald Sontheimer (at that time at The University of Alabama at Birmingham). From here, we refer to these cells as D54MG-EGFP cells, which were used in all the experiments with the exception of the immunocytochemistry data in Supplementary information, where we used unmodified D54MG cells, also provided by Dr. Harald Sontheimer [20], which originate from Dr. Darrell D. Bigner (Duke University, Durham, NC, USA). We maintained these cells in tissue culture dishes at 37 °C in a 90% air/10% CO2 atmosphere incubator in cell culture media containing Dulbecco’s Modified Eagle Medium/Nutrient Mixture F-12 (DMEM/F-12, Life Technologies, Carlsbad, CA, USA) supplemented with 2 mM L-glutamine and 7% Fetal Bovine Serum (Hyclone™, ThermoFisher Scientific, Waltham, MA, USA) and used them in the experiments within 20 passages. For the experiments, glioma cells propagating in the dishes were washed with Hank’s balanced salt solution (ThermoFisher Scientific, Waltham, MA, USA) and treated with 0.25% trypsin-EDTA (ThermoFisher Scientific, Waltham, MA, USA) for 2 mins to detach the cells. The resulting cell suspension was pelleted by centrifugation for 7 mins at 100× *g*. The pellet was resuspended in fresh culture media and plated onto round glass coverslips (12 mm in diameter) pre-cleaned with RBS™ 35 (ThermoFisher Scientific, Waltham, MA, USA) [22]. To allow cells to settle, coverslips were returned to the incubator for 1 h, after which the cell suspension was replaced with fresh cell culture media. At this point, a subset of the cells on coverslips received treatments of SWCNT-PEG or SWCNT-PEG-THFF, or the functional groups, PEG or PEG-THFF, added to the culture media and returned to the incubator until used in the experiments.

### 2.3. Live Cell Imaging

The coverslips with D54MG-EGFP cells were mounted onto an imaging chamber filled with external solution (pH 7.4), consisting of sodium chloride (140 mM), potassium chloride (5 mM), calcium chloride (2 mM), magnesium chloride (2 mM), D-glucose (5 mM) and HEPES (10 mM), at room temperature (22–25 °C) and standard atmospheric conditions. An inverted microscope (Nikon TE300; Nikon, Inc., Melville, NY, USA) equipped with differential interference contrast (DIC) and wide-field epifluorescence illumination (100 W halogen and 75 W metal halide lamps, respectively) was used with a standard fluorescein isothiocyanate (FITC) filter set to visualize the D54MG-EGFP cells. Images were acquired using a CoolSNAP®-HQ cooled charge coupled device camera (Photometrics, Tucson, AZ, USA) driven by MetaMorph® 6.1 imaging software (Molecular Devices, Chicago, IL, USA).

### 2.4. Morphometric Analysis

To assess the cellular morphology, solitary D54MG-EGFP cells were imaged, 1-day post-plating, using the above described microscope and a 60× Plan Apochromatic oil-immersion objective. The images were analyzed using a previously described algorithm [16] to measure the area and perimeter of the individual cells, except that here we used the EGFP fluorescence instead of the previously used calcein fluorescence to obtain the outline of the cell. The area and perimeter values were further used to calculate the form factor (FF; defined as [4π × (Area)/(Perimeter)^2^]) which is a measure of the roundness of an object/cell; a perfectly round/circular object has a FF = 1, while FF ≈ 0 describes a line.

### 2.5. Vitality Assay

To assess the vitality characteristics of SWCNT-treated D54MG-EGFP cells, that is, cell adhesion, proliferation and death rate, coverslips with cells were imaged 2 h and 2 days post-plating. The 2-h time point was used to establish initial plating density, that is, cell adhesion, while the 2-day time point was used to assess the proliferation of cells, under each condition. Prior to imaging, all the cells were incubated for 10 min in external solution containing 1 μg/mL of the cell permeable nuclear dye Hoechst 33342 (2′-[4-ethoxyphenyl]-5-[4-methyl-1-piperazinyl]-2,5′-bi-1H-benzimidazole trihydrochloride trihydrate; ThermoFisher Scientific, Waltham, MA, USA) to label the nuclei. After rinsing, the cells were then imaged using the above described microscope and a 20× Plan Fluor objective. The fluorescence of Hoechst 33342 was visualized using a standard 4′,6-diamidino-2-phenylindole (DAPI) filter set, while a FITC filter set was used to image the cytosol of cells, containing EGFP, with their plasma membranes intact. The FITC and DAPI images where then merged, using ImageJ software (National Institutes of Health, Bethesda, MD, USA), to visualize the cells and their corresponding nuclei. The cells positive for EGFP and Hoechst were considered live, while positively stained nuclei with Hoechst lacking surrounding EGFP stain were considered to represent dead cells. The number of nuclei per total viewed area (0.75 mm^2^) was counted, as we described in detail elsewhere [23]; cell density was expressed as number of cells per cm^2^.

### 2.6. Statistics

Statistical analysis was done using GB-STAT version 6.5 software (Dynamic Microsystems, Inc., Silver Spring, MD, USA) and SAS Software, version 9.2 of the SAS software for Windows (SAS Institute Inc., Cary, NC, USA). The number of subjects (cells or coverslips) required for comparison was estimated using power analysis (set 80% and *α* = 0.05) and guided by our previously published work [16,17,19,23]. Some groups deviated from normality based on Shapiro-Wilk or D’Agostino-Pearson tests for normality. Consequently, all the data are reported as median with interquartile range (IQR) and nonparametric statistics were used. To test the difference between the 2-h and 2-day time points in the vitality assay, the two groups were compared using Mann-Whitney U-test. For all the other experiments, the multiple independent groups were analyzed using Kruskal-Wallis One-Way ANOVA followed by Dunn’s test (significance established at *p* < 0.05).

## 3. Results

### 3.1. Effect of wsSWCNTs on the Morphology of D54MG-EGFP Glioma Cells

**Figure 1.**
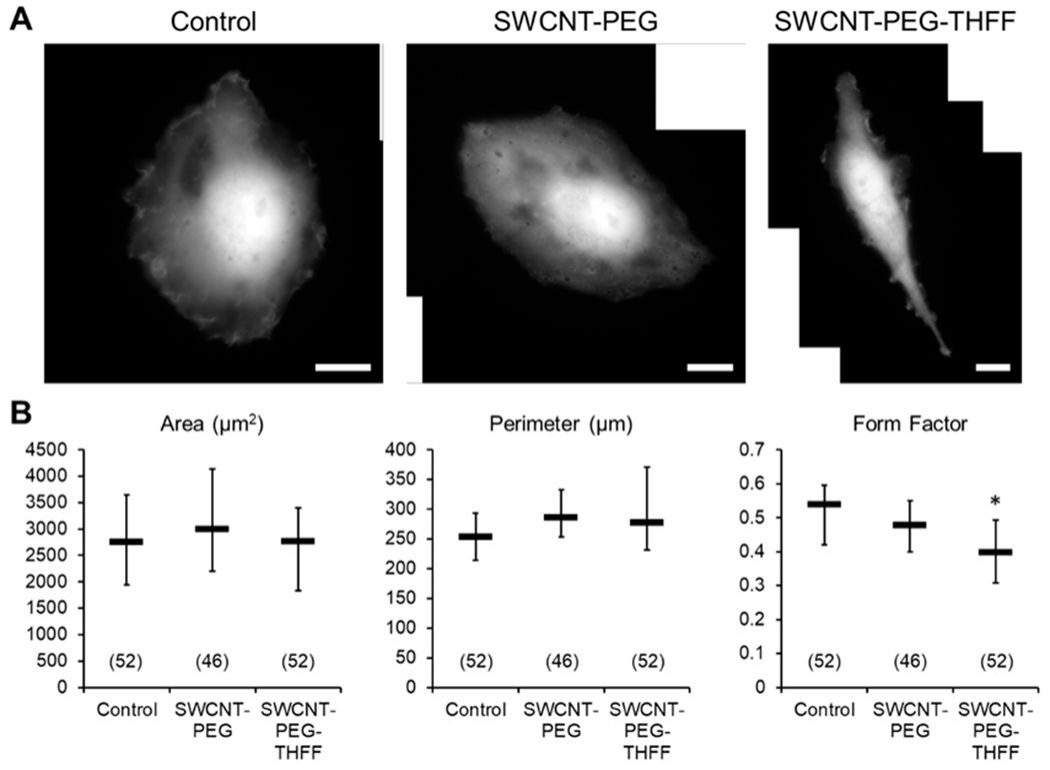
Single-walled carbon nanotubes functionalized with tetrahydrofurfuryl-tenninated polyethylene glycol (SWCNT-PEG-THFF) solute induces morphological changes in D54MG- enhanced green fluorescent protein (EGFP) human glioma cells. (**A**) Images of solitary control, SWCNT-PEG-treated and SWCNT-PEG-THFF-treated D54MG-EGFP glioma cells in culture plated onto glass coverslips. Scale bars, 20 μm. (**B**) Summary graphs showing the effects of SWCNT-PEG and SWCNT-PEG-THFF on the morphology of D54MG-EGFP human glioma cells. Number of D54MG-EGFP cells studied in each condition is given in parentheses. The boxes represent medians with interquartile range (IQR). Asterisk indicates a statistical difference when compared to the control group. Kruskal-Wallis one-way ANOVA followed by Dunn’s test. *: *p* < 0.05.

During their invasion through the extracellular space of the brain, glioma cells have to adjust their morphology and become less round [2]. Thus, we assessed the effects of wsSWCNTs on the morphological parameters of D54MG-EGFP human glioma cell line (Figure 1). To accomplish this, D54MG-EGFP cells were plated onto glass coverslips and incubated for 24 h in the absence or the presence of 5 μg/mL SWCNT-PEG or 5 μg/mL SWCNT-PEG-THFF, and then imaged using a standard FITC filter set and a 60× objective (Figure 1A). Images of solitary cells, that is, cells devoid of contact with other cells, were analyzed to obtain the area and perimeter values of the cells (Figure 1B). These values were further used to calculate the form factor, a measure of cell roundness (Figure 1B). We found that D54MG-EGFP cells, when treated with SWCNT-PEG, did not show any differences in the morphological parameters compared to the untreated cells (Figure 1B). D54MG-EGFP cells treated with SWCNT-PEG-THFF also showed no significant changes in the area and perimeter of the cells compared to the untreated cells. However, they showed a significant decrease (by ~23%) in the form factor compared to the untreated cells implying that the SWCNT-PEG-THFF causes a change in the cell shape (cells were less rounded), but not the size of D54MG-EGFP cells.

**Figure 2.**
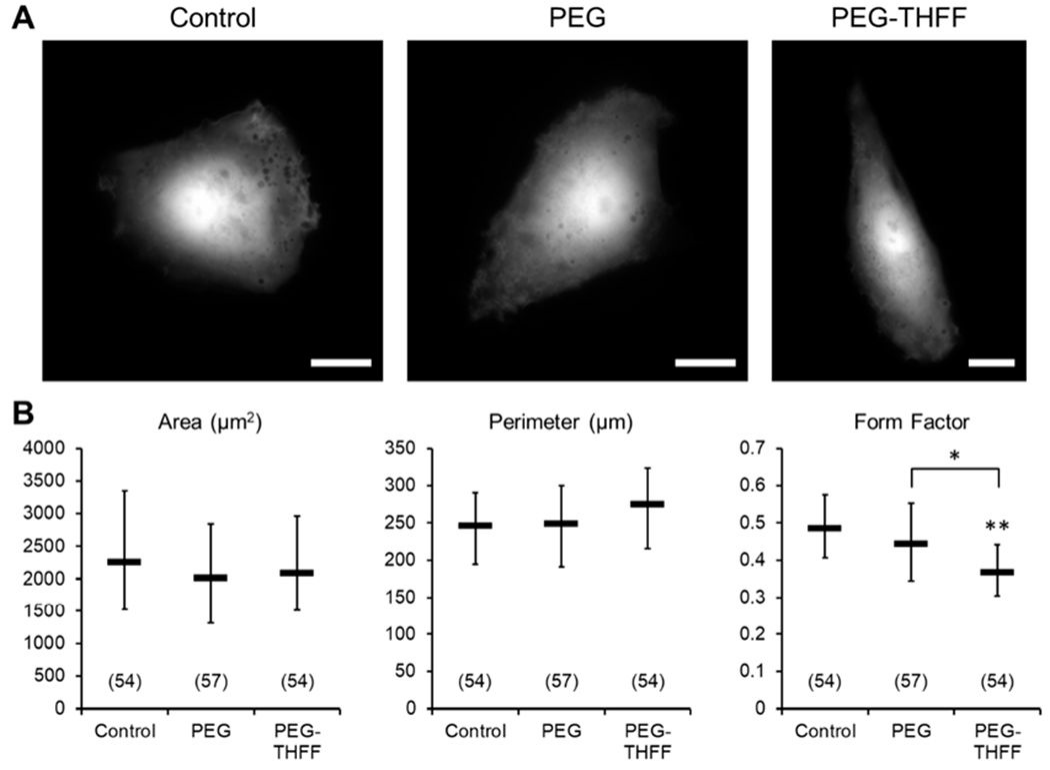
The functional group PEG-THFF induces morphological changes in D54MG-EGFP human glioma cells. (**A**) Images of solitary control, PEG-treated and PEG-TElFF-treated D54MG-EGFP glioma cells in culture plated onto glass coverslips. Scale bars, 20 μm. (**B**) Summary graphs showing the effects of the functional groups PEG and PEG-TE1FF on the morphology of D54MG-EGFP human glioma cells. Asterisks indicate a statistical difference when compared to the control group. The other difference is marked with a bracket. Other annotations as in Figure 1. *: *p* < 0.05; **: *p* < 0.01.

Since the SWCNTs have been chemically functionalized to render aqueous solubility, the question arose if the functional groups by themselves could cause any changes in the morphology of D54MG-EGFP cells. To assess this, D54MG-EGFP cells were treated with the functional groups PEG (1 μg/mL) or PEG-THFF (1 μg/mL), imaged (Figure 2A) and the morphological parameters of the cells were quantified (Figure 2B); the rationale for choosing the concentration of functional groups used is provided in Material and Methods. We found similar results to those obtained when cells were treated with wsSWCNTs, that is, the cells treated with PEG showed no significant differences in all the morphological parameters assessed and PEG-THFF-treated cells showed no significant differences in the area and the perimeter compared to the untreated cells (Figure 2B). However, there was a significant decrease (by ~24%) in the form factor of cells treated with the functional group PEG-THFF compared to the untreated cells as well to that of cells treated with PEG alone (Figure 2B). As the magnitude of this decrease in the form factor was comparable to that seen in cells treated with SWCNT-PEG-THFF (24% vs. 23%, compare Figures 1B and 2B, respectively), morphological changes observed in D54MG-EGFP cells treated with SWCNT-PEG-THFF appear to be caused by the functional group, likely its THFF moiety (as per additional comparison to SWCNT-PEG data), and not the SWCNT backbone itself.

### 3.2. Effect of wsSWCNTs on the Vitality of D54MG-EGFP Cells

**Figure 3.**
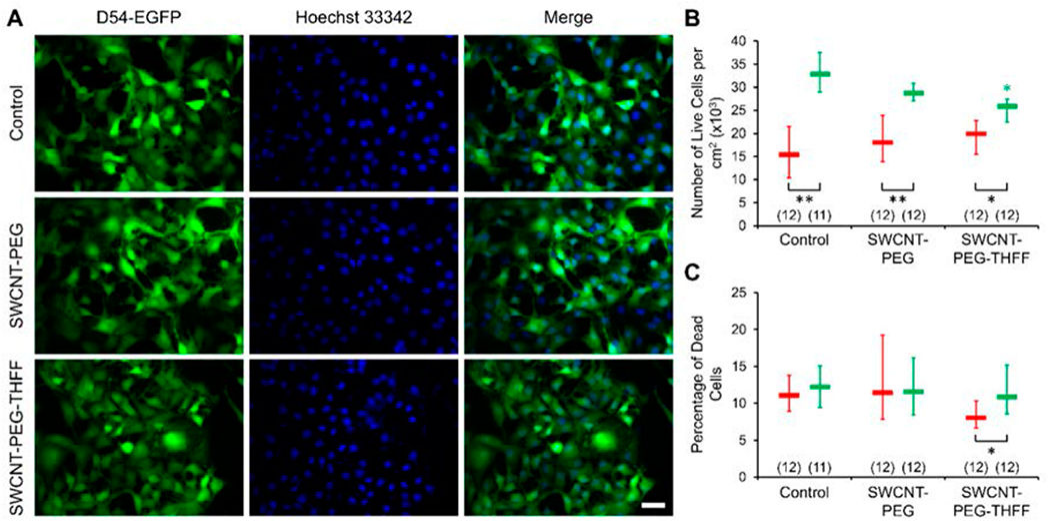
SWCNT-PEG-THFF solute reduces the proliferation rate of D54MG-EGFP human glioma cells. (**A**) Images of D54MG-EGFP glioma cells (left column) and their corresponding nuclei labeled with the cell permeable nuclear dye Hoechst 33342 (middle column); right column shows the merge of the images. Rows (top-bottom) show images of the control, SWCNT-PEG-treated and SWCNT-PEG-THFF-treated D54MG-EGFP glioma cells, 2 days post-plating. Scale bar, 50 μm. (**B**) Summary graph showing the median and IQR numbers of live cells per cm^2^ of the control, SWCNT-PEG-treated and SWCNT-PEG-THFF-treated coverslips, 2 h (red) and 2 days (green) post-plating. (**C**) Summary graph showing the median and IQR percentages of dead cells in the above setting. The numbers in parentheses represent the number of coverslips imaged in each group. Asterisks indicate a statistical difference compared to the control group. The other differences are marked by the brackets. Mann-Whitney U-test was used for the comparison between the time points and Kruskal-Wallis one-way ANOVA followed by Dunn’s test was used for the comparison between the different conditions at each time point; *: *p* < 0.05; **: *p* < 0.01.

Cell adhesion and proliferation are the factors contributing to malignancy of GBM [3,4], Thus, we studied whether wsSWCNTs cause changes in the vitality of D54MG-EGFP cells, by assessing adhesion, proliferation and death rate of these glioma cells (Figure 3). We plated D54MG-EGFP cells onto glass coverslips and incubated them for 2 h or 2 days in the absence, or the presence of 5 μg/mF SWCNT-PEG or 5 μg/mF SWCNT-PEG-THFF. The 2-h time point was used to establish the initial plating density reporting on cell adhesion, while the 2-day time point was used to assess the proliferation capability of the cells. The nuclei of the cells were labeled with a cell permeant dye Hoechst 33342. The cells were imaged using standard FITC (for cytosolic EGFP) and DAPI (nuclear Hoechst stain) filter sets and a 20× objective (Figure 3A). We quantified and reported the number of live D54MG-EGFP in each of the conditions along with the percentage of dead cells (Figure 3B,C). We found that the number of live D54MG-EGFP cells, at the 2-h time point, did not show any significant differences between the groups assessed, implying that the initial seeding density was similar across all the groups (Figure 3B). We also found that the number of live cells at the 2-day time point were significantly higher in all the groups compared to their corresponding 2-h time point implying that the cells undergo proliferation in all the conditions (Figure 3B); the proliferation rate (ratio of counts of live cells at 2-day vs. 2-h time points) was 2.1 in control, 1.6 in SWCNT-PEG-treated and 1.3 in SWCT-PEG-THFF-treated groups. However, we found that the cells treated with SWCNT-PEG-THFF show a significant decrease in the number of live cells compared to the untreated cells at the 2-day time point indicating that SWCNT-PEG-THFF reduces the proliferation rate of D54MG-EGFP cells. The percentage of dead D54MG-EGFP cells, however, did not show any significant differences between the groups at each of the time points (Figure 3C). We also did not find any significant differences between the two time points in the control and SWCNT-PEG-treated groups. However, the cells treated with SWCNT-PEG-THFF showed a significant relative increase (~35%) of dead cells at the 2-day as compared to 2-h time point (Figure 3C). Taken together, these results imply that the SWCNT-PEG-THFF solute causes a reduction in the proliferation rate of D54MG-EGFP cells along with an increase in their cell death rate.

**Figure 4.**
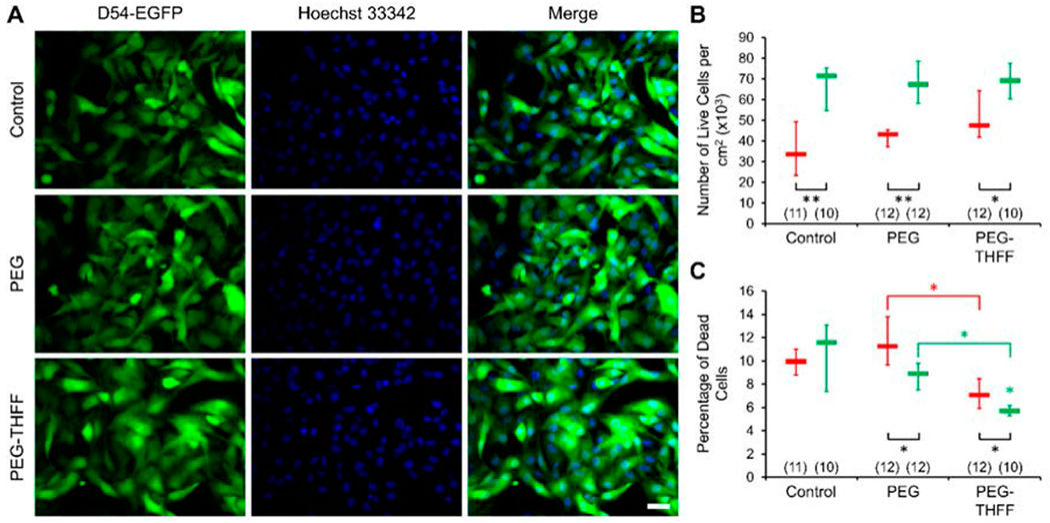
The functional groups PEG and PEG-THFF reduce the cell death rate of D54MG-EGFP human glioma cells. (**A**) Images of D54MG-EGFP glioma cells (left column) and their corresponding nuclei labeled with the cell permeable nuclear dye Hoechst 33342 (middle column); right column shows the merge of the images. Rows (top-bottom) show the control, PEG-treated and PEG-TElFF-treated D54MG-EGFP glioma cells, 2 days post-plating. Scale bar, 50 μm. (**B**) Summary graph showing the median and IQR numbers of live cells per cm^2^ of the control, PEG-treated and PEG-TElFF-treated coverslips, 2 h (red) and 2 days (green) post-plating. (**C**) Summary graph showing the median and IQR percentages of dead cells in the above setting. Other annotations as in Figure 3.

As in the morphology study, we assessed whether the functional groups by themselves could cause any change in the vitality of D54MG-EGFP cells (Figure 4). D54MG-EGFP cells were plated onto glass coverslips and incubated for 2 h or 2 days without or with the addition of the functional groups PEG (1 μg/mL) or PEG-THFF (1 μg/mL). We imaged and quantified the number of live cells in each of the conditions and time points and found that the number of live cells were not significantly different between the groups at each of the time points (Figure 4A,B). The numbers of live D54MG-EGFP cells in all the groups were significantly higher at the 2-day time point compared to the corresponding 2-h time point confirming that the cells are proliferating in all the conditions. The proliferation rates were 2.1 in control group and 1.6 in PEG-treated D54MG-EGFP cells, matching those rates obtained in SWCNT-PEG-treated cells and their controls (compare Figures 3B and 4B). The sole mismatch was a proliferation rate of 1.5 in the PEG-THFF-treated group (Figure 4B) as compared to that of 1.3 seen in SWCT-PEG-THFF-treated D54MG-EGFP cells (Figure 3B). This indicates that the effect of SWCNT-PEG-THFF on D54MG-EGFP proliferation rate seem to be mainly caused by the SWCNT backbone.

We then analyzed the percentage of dead cells and found that the cells treated with PEG or PEG-THFF did not show any significant difference in the percentage of dead cells at the 2-h time point compared to the untreated cells (Figure 4C). However, the cells treated with PEG-THFF showed significantly lower percentage of dead cells at the 2-h time point compared to the cells treated with PEG. We also found that the percentage of dead cells was significantly lower at the 2-day time point compared to the corresponding 2 h time point for the cells treated with PEG and PEG-THFF; the untreated cells did not show any difference between the two time points. In addition, we found that the cells treated with PEG-THFF showed a significantly lower percentage of dead cells compared to the untreated cells, as well as the cells treated with PEG, at the 2-day time point. Taken together, it appears that both the functional groups, PEG-THFF in particular, have a protective effect on D54MG-EGFP human glioma cells survival (Figure 4C), and that the increase death rate seen in cells treated with SWCNT-PEG-THFF is mediated by the SWCNT backbone (Figure 3C).

## 4. Discussion

In the present study, the application of SWCNT-PEG to D54MG-EGFP cells didn’t cause a change in the morphological characteristics of these malignant human astrocytes. This is in stark contrast with the results we previously obtained using primary cultures of mouse cortical astrocytes, which exhibited a reduction in the form factor upon exposure to SWCNT-PEG [16,17], That effect, associated with an increase in glial fibrillary acidic protein (GFAP) immunoreactivity [16,17], was taken as a sign of astrocyte maturation and was ascribed to the SWCNT backbone, because PEG alone had no effect on the form factor [17]. However, SWCNT-PEG failed to induce changes in the form factor of astrocytes isolated from knock-out mice lacking GFAP expression [17], pointing to a GFAP-dependent process. Interestingly, D54MG glioblastoma cells lack expression of GFAP (Supplementary Information, Figure S1), as expected for a high malignancy grade glioma [20,24]. Whether GFAP absence in D54MG cells render their irresponsiveness to the SWCNT-PEG in terms of form factor dynamics, observed in the present work, could be experimentally addressed in future by transfection of these cells to express GFAP. Similarly, the differences between the effects that SWCNT-PEG-THFF has on glioma cells, obtained here, as opposed to those it might have on primary astrocytes await further experimentation. It should be noted, however, that in the previous work on astrocytes, we plated astrocytes onto polyethyleneimine-coated coverslips, while in the present work the same kind of glass coverslips have been used plain/uncoated for the plating of D54 glioblastoma cells. As earlier studies suggested that substrate qualities play a role in neuronal [25,26] and astrocytic morphology [23], it is possible that the differential effects of SWCNT-PEG on astrocytes and D54MG-EGFP glioblastoma cells have arisen from the use of different, plain vs. coated, glass coverslips for cell plating.

The vitality assay shows that the D54MG-EGFP cells treated with SWCNT-PEG have similar adhesion, proliferation and death rate as control, untreated cells. These findings are in agreement with the results we previously obtained using wild-type GFAP-expressing astrocytes exposed to this nanomaterial (Figure 4 of [17]). However, based on that previous work, we would predict SWCNT-PEG to promote cell death rate in GFAP-negative D54MG cells. Namely, unlike their control (wild-type GFAP-expressing astrocytes), GFAP knock-out astrocytes exposed to SWCNT-PEG had a significantly increased cell death rate (Figure 4B of [17]). At the time, that finding led us to speculate that GFAP in wild-type astrocytes might have a protective role against hitherto unacknowledged harmful effects of SWCNT-PEG. The present results showing unaffected percentage of dead D54MG-EGFP cells when treated with SWCNT-PEG indicates that the very notion cannot be extended to malignant astrocytes.

In the present work, we find that PEG itself reduced the number of dead D54MG-EGFP cells (Figure 4C). This finding is at odds with our previous data collected from wild-type astrocytes showing the opposite effect, that is, increased astrocyte cell death rate in presence of PEG (Figure S2 of [17]). Thus, it appears that PEG itself is harmful to normal astrocytes, while protective of D54MG-EGFP glioma cells. These disparate finding warrants future investigation regarding the underlying molecular mechanisms that might mediate the differential effect of PEG on normal vs. malignant astrocytes. More importantly, our findings raise concerns in regard to the use of PEG itself for therapeutics in gliomas [27–29].

At present, we are unaware of a study using SWCNT-PEG-THFF on astrocytes, and hence we cannot attempt any comparison on the effects of this nanomaterial on primary astrocytes vs. their malignant counterparts. However, we compare the effects exerted by SWCNT-PEG and SWCNT-PEG-THFF on D54MG-EGFP glioma cells. The reduction of form factor seen in D54MG-EGFP cells treated with SWCNT-PEG-THFF appears to be caused by the THFF moiety of the functional group. Albeit we have not directly tested this idea, the lack of the effect on D54MG-EGFP cells form factor by PEG and SWCNT-PEG supports this inference. Whether presumed direct THFF effect on D54MG-EGFP morphology, as these malignant astrocytes become less round, is an expression of an increase in their invasiveness [2], or perhaps it represents their re-differentiation, similar to stellation/maturation in normal astrocytes [16], remains to be investigated. Vitality assay using D54MG-EGFP cells indicates that SWCNT-PEG-THFF also reduces cell proliferation and increases the cell death rate, both of which were unaffected by SWCNT-PEG (Figure 3B,C); this intuitively points again towards THFF as a mediator of the co-polymer effect on D54MG-EGFP cells. However, this idea is not supported by the effects of functional groups alone (Figure 4C), and the possible explanation is rather more complex. Namely, both functional PEG and PEG-THFF groups, without affecting cell proliferation, reduce the cell death rate (Figure 4C), an effect that is more pronounced in D54MG-EGFP cells treated with PEG-THFF. Taken together, it appears that the functional groups we use are protective for D54MG-EGFP cells, while the SWCNT backbone seems detrimental to these malignant human astrocytes. It is tempting to speculate that the differential effect of SWCNT-PEG and SWCNT-PEG-THFF is due to an increase in the bioactivity of SWCNT-PEG-THFF, likely due to the utilization of THFF, which is known to prevent possible PEG cross-linking between SWCNT-PEG [18] and thus increases water solubility of SWCNT-PEG-THFF and its bioactivity. Consequently, any use of SWCNT co-polymers in therapeutic approaches for gliomas will need to be preceded by the synthesis and characterization of SWCNT copolymers utilizing some functionalization group(s) that would not oppose the SWCNT harmful effect. Given the multitude of chemistries and functional groups available for carbon nanotubes [5], this task seems doable, albeit time-consuming. Moreover, our data raise concerns over the use of THFF groups to enhance bioavailability of variety of compounds (e.g., thiamine) in the brain for in vivo clinical translational applications [30,31].

Interestingly, Santos et al. [10] used carboxylated SWCNTs (SWCNT-COOH) on human U251 glioma cell line. They found that U251 cells had reduced proliferation rate by ~20% upon a 3-day treatment with SWCNT-COOH at the concentration of 3 μg/mL. Furthermore, they observed an increased cell death rate (~36% for apoptotic death rate and ~170% for necrotic death rate; estimated from their Figure 1A) at the concentration of 10 μg/mL [10]. These findings are similar to those we observe when treating D54MG-EGFP cells with SWCNT-PEG-THFF, with the proliferation rate reduced by ~21% (control vs. SWCNT-PEG-THFF; Figure 3B) and the cell death rate percentage increase by ~35% (2-h vs. 2-day time period for SWCNT-PEG-THFF group; Figure 4C). It should be noted that we use SWCNT-COOH (albeit from a different source than those used in Santos et al.) as an initial reactant, which gets completely consumed, in the synthesis of both SWCNT-PEG and SWCNT-PEG-THFF (details available in [16,18]). As the treatment of U251 glioma cells with the COOH functional group was not reported [10], we cannot make further comparisons to our data. Also, the comparison made above is based on two different glioma cell lines grown on two different strata and under different culturing conditions; U251 cells were grown on a plastic stratum [10], while here D54MG-EGFP cells grew on plain glass coverslips. However, these technicalities should not distract from the emerging picture that, depending on conditions and cells treated with SWCNT conjugates, it appears as both the SWCNT backbone and/or functionalized groups, albeit the latter only meant to increase water solubility of SWCNT conjugate, can exert a biological effect.

In the context of glioma SWCNT therapeutics, our present work indicates that additional care should be taken in the selection of functional groups, as SWCNT conjugates may cause differential biological effects mediated by the SWCNT backbone and/or functional group.

## Supplementary Material

1
