# Supplementary material for: Effects of Chemically-Functionalized Single-Walled Carbon Nanotubes on the Morphology and Vitality of D54MG Human Glioblastoma Cells
Source: Neuroglia. Author manuscript; Available in PMC 2019 May 15. (PMC6519950; doi:10.3390/neuroglia1020022)
Supplement: 1 [file NIHMS993470-supplement-1.pdf]

neuroglia

Article

Effects of Chemically-Functionalized Single-Walled Carbon Nanotubes on the Morphology and Vitality of D54MG Human Glioblastoma Cells

Seantel Hopkins, Manoj K. Gottipati, Vedrana Montana, Elena Bekyarova, Robert C. Haddon, Vladimir Parpura

This file includes:
Methods and Materials
Supplementary Figure S1
References

Materials and Methods

Here we only provide an essential summary of the methods used in the generation of Supplementary Figure S1.

Purified astrocytic cultures. Astrocytes isolated from the visual cortices of 0-2-day-old C57BL/6 (wild-type) mice were purified and maintained in cell culture as we previously described [1,2]. Experiments were conducted on purified astrocytes grown on polyethyleneimine (PEI)-coated glass coverslips. Experimental protocols were approved by Institutional Animal Care and Use Committee at the University of Alabama at Birmingham.

Glial fibrillary acidic protein immunocytochemistry. Astrocytes and D54MG glioma cells grown on PEI-coated and uncoated glass coverslips, respectively, were fixed with freshly prepared Dent's fixative (80% methanol and 20% dimethyl sulfoxide) and labeled for glial fibrillary acidic protein (GFAP) using a primary mouse monoclonal antibody (1:500 dilution; ICN Cat. No. 69110; MP Biomedicals; Solon, OH) followed by a tetramethylrhodamine isothiocyanate (TRITC)-conjugated secondary antibody [2]. After completion of the labeling procedure, the cells were analyzed for the density of GFAP immunoreactivity based on the images acquired using a standard TRITC filter set and a 60× Plan Apo objective [1]. The cell area was established by manual tracing of the differential interference contrast (DIC) images [1].

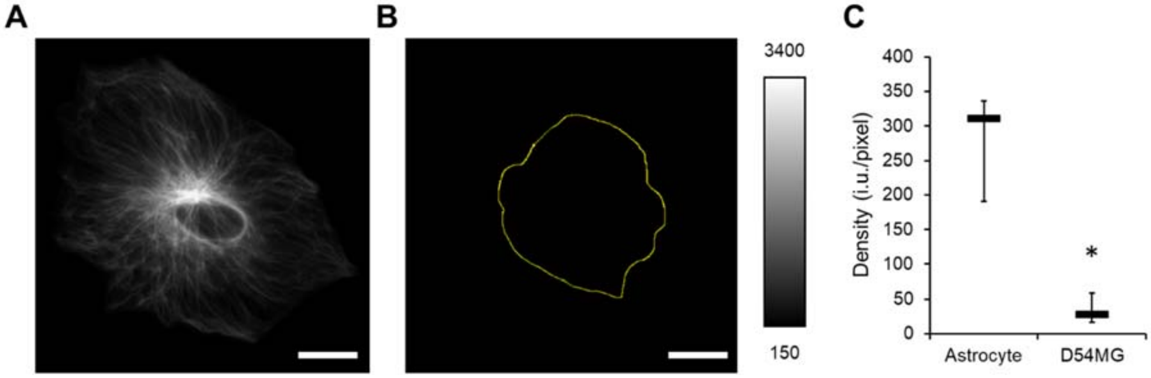

Supplementary Figure S1. Glial fibrillary acidic protein (GFAP) expression in primary mouse astrocytes and lack thereof in D54MG human glioma cells. A) GFAP immunoreactivity (ir) of a solitary astrocyte. B) Lack of GFAP expression in solitary D54MG human glioma cell, whose perimeter is traced in yellow based on the corresponding DIC image. Gray scale is a linear representation of the fluorescence intensities, expressed in fluorescence intensity units (i.u.), of the pixels in the images. Scale bars, 20 μm. C) Summary graph comparing the density of GFAP-ir, in astrocytes and D54MG human glioma cells (n=5 in each group). The boxes represent medians with interquartile ranges. Asterisk indicates a significant difference determined by Mann-Whitney U-test. *: $p<0.05$.
